# Effectiveness of mobile health based vision screening on uptake of referral services in school children: A randomized control trial

**DOI:** 10.12669/pjms.41.8.11742

**Published:** 2025-08

**Authors:** Ayesha Javed, Shamaila Mohsin, Ramsha Habib, Uzma Ahsan Malik

**Affiliations:** 1 Ayesha Javed, MBBS, MPhil. Department of Community Medicine and Public Health, Army Medical College, National University of Medical Sciences, Rawalpindi, Pakistan; 2 Shamaila Mohsin, PhD, MHPE, MPhil, MPH, MBBS, Dip (Med Adm) Associate Professor, Department of Public Health, AFPGMI, Rawalpindi, Pakistan; 3 Ramsha Habib, MBBS, MPhil. Department of Community Medicine and Public Health, Army Medical College, National University of Medical Sciences, Rawalpindi, Pakistan; 4 Uzma Ahsan Malik, MBBS, TDRCPCH Pediatrics. Department of Pediatrics Medicine, Hameed Latif Hospital, Lahore, Pakistan

**Keywords:** mHealth intervention, mHealth reminder, Preventable blindness, Referral uptake, Visual impairment, Vision screening

## Abstract

**Objective:**

To assess the effectiveness of mobile health (mHealth) based vision screening and automated multicomponent SMS reminders on referral uptake among school children in a low resource setting.

**Methodology:**

This was a concurrent parallel group; single blinded, randomized control trial. The participants were first screened for visual impairment using mHealth visual acuity app. Then schools were randomly allocated (1:1) to an intervention and control group. Children 5-15 years old with parents having smart phone and diagnosed with visual impairment on screening were eligible. A multicomponent SMS reminder in addition to printed referral form was given to the intervention group. It contained a health promotion message, visual depiction of child’s vision along with customized action plan. SMS was delivered through visual acuity application with automated SMS software. Referred children were followed for eight weeks through telephonic follow up. Data was analyzed according to intention to treat principle with Cox regression model to analyze the effect of SMS reminder on referral uptake.

**Results:**

A total of 320 children were enrolled in this study. Visual impairment was identified in 82 children (25.6%). At eight weeks post intervention the proportion of children who attended the hospital was significantly higher in the intervention group compared to the control group, 73.8% vs. 37.5%, [Hazard ratio (HR) 2.428; (95% CI 1.26-4.675); p=0.008]. About 9 (10.9%) enrolled participants were lost to follow up. There was no statistically significant association between hospital attendance among referred children and their sociodemographic characteristics.

**Conclusion:**

mHealth referral reminder effectively improves the uptake of referral services following vision screening among school children as the percentage of children who attend the hospital was significantly increased in the intervention group.

**Trial registration:**

ClinicalTrials.gov ID NCT06616051

## INTRODUCTION

Globally there are over two billion individuals with visual impairment.^1^ Almost two third of this burden of disease is situated in low to middle income countries (LMIC).^2^ A significant proportion of those affected with visual impairment are children.^3^

Pakistan ranks third among top twenty countries affected by blindness or moderate to severe visual impairment.^4^ In Pakistan more than two million children are estimated to be affected from blindness and visual impairment.^5^

Evidence has indicated that visual impairment (VI) significantly diminishes quality of life especially in young children and becoming a prominent public health concern worldwide.^6^ Childhood visual impairment creates obstacles to learning, growth and educational achievement which in turn impacts future employment opportunities and socioeconomic standing.^7^

A recent systematic review (2000-2020) reported that uncorrected refractive errors especially myopia is the most prevalent cause of vision impairment among children.^8^ Evidence suggests that possible developmental delays occur when refractive problems are not corrected in infancy and early childhood.^9^ Uncorrected refractive problems in this age range also have a correlation with clinically acknowledged deficits in cognitive and visual-motor skills.^10^ In addition, it may result in both immediate and long-term negative physical and psychological effects, such as increased susceptibility to accidents and injuries, poor visual-motor skills, low self-esteem, depression and anxiety, and verbal and physical bullying.^11^

In terms of time and resources, school eye health programs are efficient.^12^ and provide a cost-effective paradigm for providing eye care to school children.^13^. Evidence suggests that timely intervention such as vision screening carry potential to prevent the vicious circle of personal and social decline among children.^14^ However, one of the key challenges highlighted on evidence regarding visual screening programs is the low uptake of referral services for children diagnosed with visual impairments, which significantly limits the effectiveness of these programs.^15^

mHealth has emerged as one of the most significant public health interventional tools in industrialized nations and is rapidly being adopted in many public health programs in LMICs.^16^ Numerous mobile health organizations have created smartphone apps that screen for visual acuity, make it easier to refer patients to eye care professionals, and notify those who have been screened.^17^ Evidence indicates that mHealth has shown promise in Pakistan, particularly in improving chronic disease management, such as diabetes care, medication adherence in stroke survivors, and increasing immunization uptake.^16^

There is scarcity of evidence regarding association between the use of mHealth and improvement of referral uptake in LMICS has been shown to date. Furthermore, there have been recommendations for additional research in LMICs to demonstrate a strong causal association between the effectiveness of m-health in improving referral uptake following a screening program.^18^ To the best of our knowledge, this research is the first of its kind a multi-component approach related to professional guidelines, recommendations, and technology application, that has not been utilized in this way in preexisting literature. The aim of the study was to assess the benefit of mHealth reminders in improving the uptake of referral services, and consequently enhancing the effectiveness of vision screening.

## METHODOLOGY

A concurrent parallel group, single blinded randomized control trial was conducted to assess the effectiveness of a multicomponent SMS reminder on referral uptake. The school were randomly assigned in a 1:1 allocation ratio using computerized randomization into two parallel groups with one group receiving conventional method of referral (printed referral form) while the parallel group receiving multi component SMS reminders in addition to printed referral form. The trial was registered with clinical trial registry prior to the recruitment of participants (ClinicalTrial.gov ID NCT0661605, Dated 26 September 2024). The trial was carried out following the CONSORT statement.

The study was performed in two federal government (FG) schools of Rawalpindi district, Pakistan. FG schools cater to the educational needs of majority of Pakistani children and students mostly studying in these schools belong to low to middle income families.

### Ethics approval:

All children participating in the trial were required to provide written informed consent from parents or guardians to permit them to participate in the research. Informed assent was obtained from children prior to the commencement of screening. Consent forms were provided in native language (Urdu) to ensure clear understanding. Special consideration was given to the timing of messages to avoid causing discomfort, such as avoiding late-night texts. The messages contained no personally identifiable information, and the messaging system was securely maintained at a single site with limited access. A contact number was also provided for participants to address any questions or concerns.

### Ethical Approval:

Ethical approval was obtained from Ethical Review Committee of Army Medical College (ERC/ID/415, Dated 05 September 2024). Approval for the conduct of screening tests was granted by FG Education Institutions Directorate Rawalpindi (0409/F-28/2024-FGEI (CPD-Trg), Dated 20 October 2024).

Study participants were 5-15 years of age as per WHO recommendations for vision screening^19^, male and female (in equal proportion) detected with visual impairment through mHealth based vision screening. Participants were recruited from two FG schools. School-A had a total enrollment of 1250 students with 860 (M=450, F=410) were 5–15 years of age while School-B had a total enrollment of 1300 students with 790 (M=400, F=390) were 5–15 years of age, studying in Grade-1 to Grade-9.

### Inclusion Criteria:

Children were eligible if they were aged between 5 and 15 years and had been detected with visual impairment during screening. Additionally, parent in possession of or have access to smartphone and have the ability to receive, read, and understand an SMS.

### Exclusion Criteria:

Children were excluded if they had a known refractive error such as myopia, were already wearing spectacles, or had any apparent eye diseases (e.g., conjunctivitis, red eye, ocular trauma, trachoma, etc.). Children whose families planned to travel outside the city within the two months following enrollment were also excluded.

A meeting with the principal of the each FG schools was held to discuss the purpose of the research. A promotional speech was given in the school to inform parents and pupils about the importance of screening. Children enrolled in the study were assessed for eligibility, and those who provided assent and had parental consent underwent visual acuity testing using the validated Peek Acuity app. This app, which includes a comprehensive software platform with data collection capabilities and SMS reminder functionality, was used on a smartphone. The Peek Acuity app has demonstrated a sensitivity of 84.6%, specificity of 97.7%, positive predictive value of 68.8%, and negative predictive value of 99.1%.^20^,^21^ Distance visual acuity cut-off was four out of the five optotypes at 6/12 level at a distance of three meters. Vision screening was carried out by two researchers (RH and UAM), assisted by three school teachers. Visual acuity testing was followed by the randomization to either the intervention group or control group.

**Fig.1:**
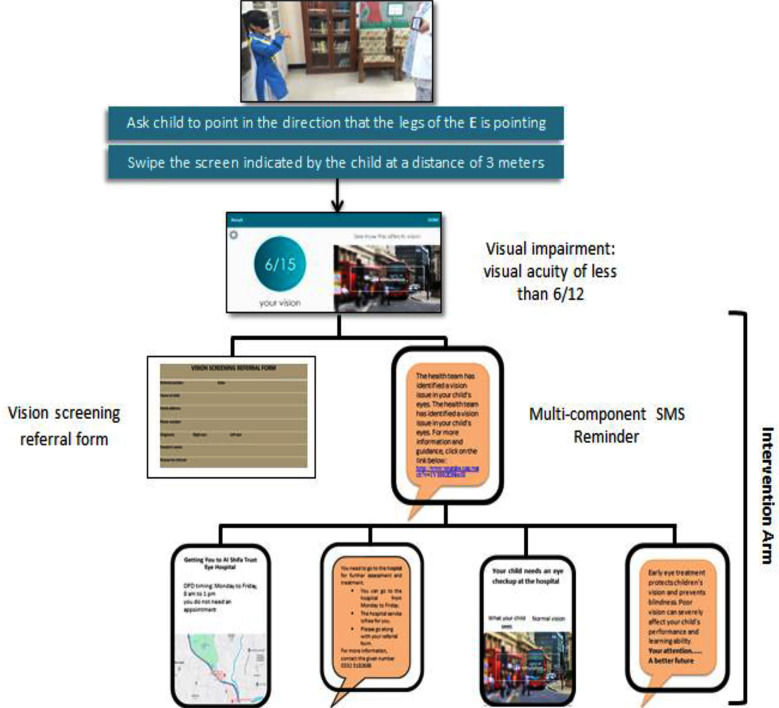
Information flow diagram.

In the intervention group parents or guardians of children received a multi-component SMS reminder in addition to the printed referral forms. These reminders were delivered every Sunday, Tuesday, and Thursday, for a duration of eight weeks or until child visited the hospital. Research team developed an automated text message reminder system for this study, taking into account the local context. The message was framed with assistance from a panel of public health consultant and research supervisors and drew on resources from the WHO Health Promotion and Disease Prevention Messages Library.^18^ The SMS reminder system comprised two main components: a) web-based application for children registration and automated reminder scheduling, and b) an SMS application for text messaging that is sent automatically. Text messages sent in Urdu, the native language, and did not require a reply. The message included four elements: Health promotion message, visual depiction of a child’s vision, a customized action plan detailing how to get to the hospital including the location, how much money is needed and what to carry with them (Fig.1). Intervention was delivered by principle investigator (AJ) under strict supervision of research supervisor (SM).

In the control group participants were provided a printed referral form. The parents of visually impaired children were guided to seek further assessment and treatment. Additionally, parents were informed of the scheduled follow-up date, which was set for every Friday for eight weeks.

The participants were required to follow up every week. A coding list of screened positive children, along with their parents’ contact numbers was provided to an independent researcher appointed to carry out follow-up. Researcher was masked to the group that the participant was assigned. Telephonic follow-up was conducted weekly for eight weeks to monitor the proportion of referred children who attended the hospital in both the groups. The parent who didn’t reply the phone call was marked in the list and contacted again up to three times. Additionally the filled in referral forms were submitted back to the teacher and collected by the research team (AJ and RH). Outcome assessment was performed by a separate independent researcher who was also masked to the assigned groups. Enrollment began on 21^st^ October 2024 and last participant was recruited on 26^th^ October 2024. The last follow up was conducted on 20^th^ December 2024.

Sample size was calculated using Clinical Calculator (https://clincalc.com/stats/samplesize.aspx) for sample size calculation with ∞ = 0.05 and Power = 80, sample size was found to 72. The referral uptake reference value 54% Vs 22% was determined by a previous study^22^. Adjusted sample size with 10% expected dropouts = sample size (1+dropout rate) = 80. For enrollment we applied a population proportion of 21.8% (with 20% non- response rate) from a regional study^23^ and found the number of 319. This is the number of school children need to be screened to obtain the required sample size of visually impaired children, eligible for the study.

Pilot testing was conducted on 10% of the intended sample size, comprising eight participants. The purpose was to evaluate the smooth implementation of the intervention and to identify any systematic errors. These participants were not included in the final analysis. The pilot sample was in addition to the 80 participants who were ultimately enrolled in the main study.

Computerized random number generator program https://www.sealedenvolope.com/ was used to allocate schools in 1:1 ratio. An independent resident from community medicine department of Army Medical College was assigned to make cards for random assignment of the allocated schools. The cards were sealed in opaque envelopes with sequential numbers. The allocation sequence was concealed from the research team (RH, UAM) responsible for participant enrollment and assessment. After allocation, the principal investigator (AJ) wrote the school’s name on each envelope to prevent subversion of the random allocation.

It was not possible to blind the study participants or data collectors due to the nature of the intervention. However, during the course of the trial, the assigned person who conducted follow-up and data analyst remained blinded.

### Primary outcome measure:

Primary outcome of interest was the proportion of referred children who attended the hospital within eight weeks of referral in both groups. It was assessed by weekly telephonic follow up of referred children.

### Secondary outcome measures:

Secondary outcome of interest was the association of hospital attendance with sociodemographic characteristics of referred participants and was assessed by comparing the sociodemographic characteristics of referred participants who attended the hospital with those who did not, using follow-up data.

### Statistical analysis:

**Fig.2:**
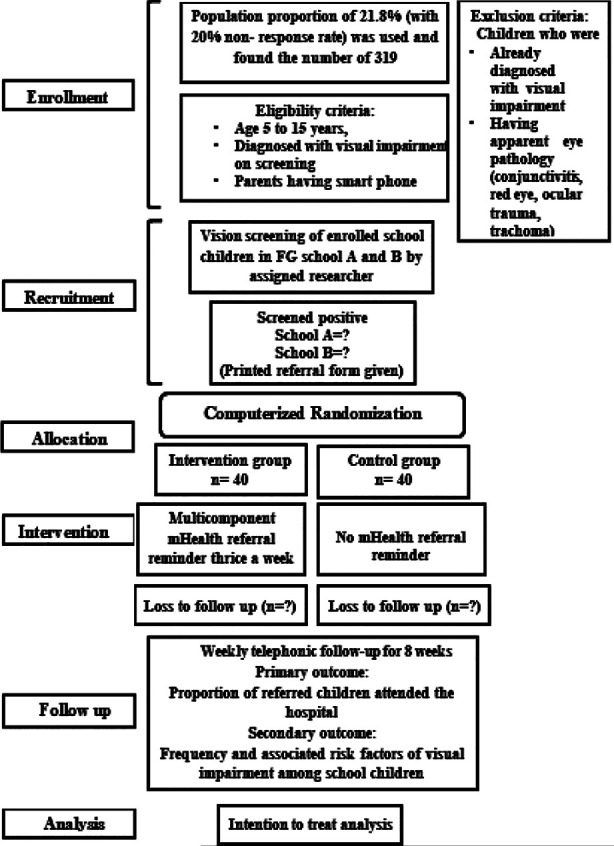
Flow chart of algorithm of trial steps.

Data was analyzed using SPSS 27 and checked for normality by applying Kolmogorov-Smirnov (KS) test. A P value of 0.05 or less was considered significant. Percentages were reported for sociodemographic characteristics, frequency of visual impairment among study participants and the number of referred children who attended the hospital. Analysis was performed using the intention to treat principle. Cox regression model was used to analyze the effect of referral reminder on referral uptake using hazard ratios with 95% confidence intervals (CIs). Statistical conclusions were based on the significance of estimated hazard ratios. We also used Kaplan-Meier estimates of the cumulative proportion of children achieving an event (referral uptake) to graphically illustrate the trial results. To overcome the missing data (attrition), adjusted sample size with 20% dropout was used and data analyzed according to “Intention to treat” principle (Fig.2).

## RESULTS

**Fig.3:**
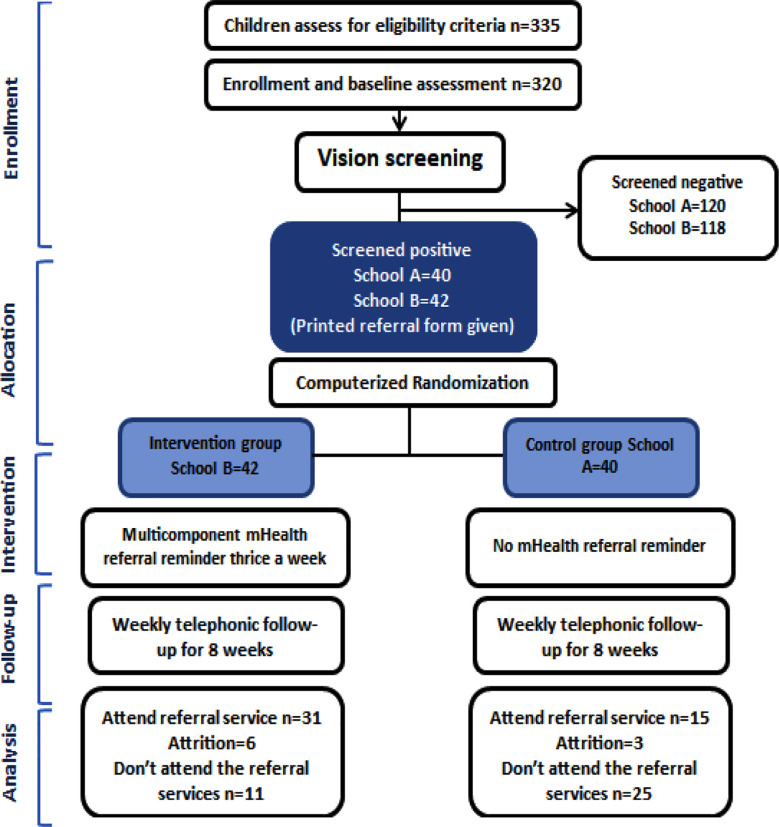
CONSORT Flow chart.

Three hundred thirty five children were assessed for enrollment where 15 were initially excluded due to ineligibility or lack of consent while other 238 were excluded because they had normal vision on screening. School-B with 42 (50.1%) visually impaired children was randomized to intervention group, while school-A with 40 (49.0%) visually impaired children in control group. After eight weeks, six were lost to follow up in intervention group while three in control group (Fig.3). Visual impairment was identified in 82 children (25.6%), with 35 (42.68%) males and 47 (57.3%) females (Table-I).

**Table-I:** Frequency of visual impairment (n=320)

	Visual impairment				
	Male		Female		Total
	Frequency	Percentage %	Frequency	Percentage %	
FG school (A)	18	45.0	22	55.0	40(49%)
FG school (B)	17	41.0	25	59.0	42 (51%)
Total	35 (42.68%)		47 (57.31%)		82 (25.6%)

A total of 82 participants were analyzed in the study (42 in intervention group and 40 in control group). Of these, 35 (42.7%) were male while 47 (57.3 %) were female. The gender distribution was relatively same between the groups, with a slightly higher proportion of females in the intervention group (59.0%) compared to the control group (55.0%). A greater proportion of fathers in the control group had attained secondary education (53.8%) compared to the intervention group (41.9%). Similarly secondary education was slightly more prevalent among the mothers in the control group (48.7%) than in the intervention group (41.9%). Lastly higher percentage of families in the intervention group reported low income (60.4%) compared to the control group (53.8%). These differences were not statistically significant (Table-II). Among the 42 children referred from the intervention group, 31 (73.8%) attended the hospital during the 8-week follow-up period, compared with 15 (37.5%) of the 40 children referred from the control group (Table-III).

**Table-II:** Sociodemographic characteristics of screen positive children.

	Combined (n=82)		Intervention group (n=42)		Control group (n=40)		p-value
	n	%	n	%	n	%	
***Gender***							
Male	35	42.7%	17	41.0%	18	45.0%	0.874
Female	47	57.3%	25	59.0%	22	55.0%	
***Father education***							
None	9	10.9%	5	11.6%	4	10.3%	
Primary	34	42.5%	19	46.5%	15	35.9%	0.546
Secondary	39	47.5%	18	41.9%	21	53.8%	
***Mother education***							
None	10	12.2%	6	13.9%	4	10.2%	
Primary	35	43.7%	18	44.2%	17	41.0%	0.783
Secondary	37	45.1%	18	41.9%	19	48.7%	
***Household Income***							
Low-Less than PKR 42000	47	57.3%	26	60.4%	21	53.8%	
Lower middle-PKR 42000-99999	30	36.6%	13	32.5%	17	41%	0.715
Middle-PKR100000-149000	5	11.9%	3	6.9%	2	5.1%	

**Table-III:** Proportion of children who referred to hospital and proportion who presented to hospital.

	Intervention Group		Control Group	
Children with visual impairment on screening referred to hospital				
	Frequency	Percentage%	Frequency	Percentage%
No of Children	42	51.0	40	49.0
Male	17	41.0	18	45.0
Female	25	59.0	22	55.0
***Children with visual impairment on screening who presented at hospital***				
	***Frequency***	***Percentage%***	***Frequency***	***Percentage%***
No of Children	31	73.8	15	37.5
Male	13	41.9	7	46.6
Female	18	58	8	53.3

The follow-up time; that was divided into weekly sections and hazard ratio (HR) compares the likelihood of referral uptake between the intervention and control groups over time. This estimation was only possible for the first five weeks of follow-up, as no children from the control group attended the hospital after weeks five. A hazard ratio greater than one indicates that the intervention group had a higher rate of hospital attendance compared to the control group, with statistical significance in several weeks. The intervention group demonstrated approximately a twofold increase in the likelihood of hospital visits compared to the control group, as revealed by univariate Cox regression model [80.5% vs. 36.3%, hazard ratio [HR] 2.247; (95% CI 1.21- 4.175); p = 0.010 on log-rank test] (Table-IV). Similar results were obtained when model was adjusted for age, gender, educational status of parents and household income [HR 2.428; (95% CI 1.26- 4.675); p = 0.008] (Table-V).

**Table-IV:** Hazard ratio of hospital attendance among children after referral during each week of the trial.

Time	Intervention Group (n %)	Control Group (n %)	Hazard ratio (95% CI)	p-value
Week 1	6 (14%)	5 (13%)	1.893 (1.022-3.507)	0.037
Week 2	8 (33%)	4 (23%)	2.010 (1.085-3.724)	0.001
Week 3	5 (45%)	3 (31%)	2.068 (1.116-3.832)	0.021
Week 4	5 (57%)	2 (36%)	2.128 (1.147-3.947)	0.017
Week 5	4 (66%)	1 (38%)	2.158 (1.163-4.006)	0.015
Week 6	0 (66%)	0 (36.3%)	----	---
Week 7	2 (70%)	0 (36.3%)	----	---
Week 8	1 (72%)	0 (36.3%)	----	---

**Table-V:** Crude and Adjusted Hazard ratio of hospital attendance among children.

Time (week)	Intervention Group (n %)	Control Group (n %)	Crude Hazard ratio (95% CI)	Adjusted Hazard ratio (95% CI)
8 Weeks	29 (80.5%)	11 (36.6%)	2.247 (1.21-4.175)	2.428 (1.26-4.675)

**Fig.4:**
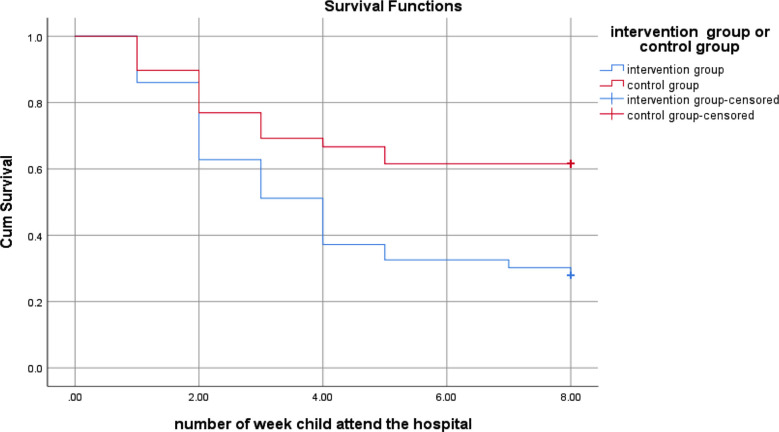
Kaplan-Meier analysis of time from screening to attendance at the hospital.

The Kaplan-Meier curve also demonstrated a clear difference in referral uptake rate between the two groups (Fig.4). Here time is plotted on the x-axis in weeks and the survival rate (estimated probability) is plotted on the y-axis. Children, who did not attend the hospital till the end of 8 week or lost to follow up during 8 week of follow-up period, were censored in the model. A steeper slope indicates a higher event (referral uptake) rate. A flatter slope indicates a lower event rate while plateaus or flat areas, indicating no event occur. The intervention group curve showed a steeper decline at each time point (a faster decline in survival probability), indicating a higher rate of referral uptake as compared to the control group. Furthermore the control group curve plateaued after week five, reflecting no further hospital attendance.

Bivariate and multivariate logistic regression analyses revealed no statistically significant associations between hospital attendance among referred children and their sociodemographic characteristics (Table-VI).

**Table-VI:** Bivariate and Multivariate Logistic Regression output of factors associated with referral uptake.

	Hospital attendance		COR (95% CI)	AOR (95% CI)
	Yes (n=46)	No (n=36)		
***Gender***				
Male	20	15	1.077 (0.446-2.603)	1.008 (0.396-2.569)
Female	26	21		
***Father educational status***				1.888(0.396-2.569)
None	06	03	1	
Primary	17	17	1.391 (0.303-6.398)	
Secondary	23	16	0.696 (0.275-1.758)	
***Mother educational status***				
None	04	06	1	
Primary	20	15	0.455 (0.109-1.890)	2.436(0.535-11.13)
Secondary	22	15	0.909 (0.356-2.321)	
***Household Income***				
Low-Less than PKR 42000	28	19	1	
Lower middle-PKR 42000-99999	15	15	0.982 (0.150-6.449)	1.096(0.154-7.820)
Middle-PKR100000-149000	03	02	0.667 (0.097-4.579)	

## DISCUSSION

Our study provides evidence that mobile health-based vision screening and automated multicomponent SMS reminders can effectively and significantly enhance the referral uptake based in a low resource setting. This study contributes important data on childhood vision health in low-to-middle-income communities (LMICs) by reporting the frequency of visual impairment among school children, in contrast to previous regional studies. It demonstrates a significant increase in follow-up rates, highlighting the potential of mobile health (mHealth) to bridge healthcare gaps and ensure timely intervention for children at risk of permanent visual impairment. To the best of our knowledge, this is the first ever trial conducted in Pakistan which assess the effectiveness of an mHealth intervention to improve referral uptake following vision screening.

Our results are consistent with a similar study carried out in Kenya^22^ and Iran^24^, where a substantial increase in hospital attendance, demonstrating the particular benefits of digital tools for underserved and low-income populations. The similarity in results is due to widespread mobile access and common barriers like poor compliance, low health literacy, and weak referral systems, that digital intervention effectively address.

However this contrast with previous findings from several studies carried out in Pakistan^25^, Nigeria^26^ and Kenya^27^ where SMS reminders have limited impact on improving referral uptake or adherence. The limited impact may be attributed to several factors, including high baseline levels of adherence, economic constraints, systemic healthcare barriers, and the absence of supportive infrastructure.

mHealth based screening tools represent an emerging innovation with substantial potential to enhance health outcomes. Our study demonstrated significant improvements in early detection, particularly in resource-limited settings, where such tools prove especially effective. The result shows a notable prevalence of visual impairment among school children compared to previous regional studies. For instance, a study conducted in 2016 reported a prevalence of 12.4.^28^ The 2019 national survey in Pakistan reported a prevalence of 15.38%, which projected a rise in the coming years.^4^ International studies, including those from South East Africa^29^,^30^ and Egypt^31^, have reported varying prevalence rates ranging from 16% to 24.7% further highlighting the variability of visual impairment rates across different regions and populations.

The disparity could be attributed to a variety of factors, including differences in study design, the target population, and the specific inclusion criteria used. Economic disparities, ethnic differences, and access to healthcare services likely play a significant role in influencing the prevalence of visual impairments.^32^ Moreover, demographic factors such as age, gender, and race can contribute to variations in the prevalence of visual impairment.^33^

### Strength of our study:

The major strength of our study is the gold standard RCT design with allocation concealment, outcome ascertainment, effort to reduce attrition and Robust statistical approach which enhancing the credibility of the study. This study adds to the growing body of evidence by showing that mHealth can also improve referral uptake related to eye care and could be incorporated as standard practice to enhance service utilization. Furthermore the findings are especially timely, aligning with global initiatives such as VISION 2020: The Right to Sight (WHO, 2023), which prioritize improving access to eye care services.

### Limitations:

In addition several limitations must be considered when interpreting the findings. The follow-up period for this study was limited to eight weeks, which may not fully capture the long-term impact of mobile health interventions on referral uptake rate. Although mobile phone text messages are widely used as mHealth intervention, one limitation of this study is that it relied solely on SMS reminders. Given the high rates of illiteracy in Pakistan, it is possible that many parents or guardians could not fully comprehend the text messages, potentially reducing the intervention’s effectiveness. Variations in network access and mobile literacy could affect the generalizability. Longitudinal studies would be better examining the causal relationships between risk factors and visual impairment.

## CONCLUSION

mHealth referral reminder effectively improves the uptake of referral services following vision screening among school children as the percentage of children who attended the hospital was significantly increased in the intervention group.

A comprehensive assessment of teachers and health workers should be conducted to evaluate their digital literacy, familiarity with mobile technology, and ability to effectively use vision screening applications. It is also recommended to assess the community’s readiness and cultural acceptance of mHealth technology to ensure that the tools and communication methods are culturally appropriate, linguistically accessible and aligned with local believes.
